# *Nf2*/Merlin Controls Spinal Cord Neural Progenitor Function in a Rac1/ErbB2-Dependent Manner

**DOI:** 10.1371/journal.pone.0097320

**Published:** 2014-05-09

**Authors:** Cynthia Garcia, David H. Gutmann

**Affiliations:** Department of Neurology, Washington University School of Medicine, St. Louis, Missouri, United States of America; IBMC - Institute for Molecular and Cell Biology, Portugal

## Abstract

**Objective:**

Individuals with the neurofibromatosis type 2 (NF2) cancer predisposition syndrome develop spinal cord glial tumors (ependymomas) that likely originate from neural progenitor cells. Whereas many spinal ependymomas exhibit indolent behavior, the only treatment option for clinically symptomatic tumors is surgery. In this regard, medical therapies are unfortunately lacking due to an incomplete understanding of the critical growth control pathways that govern the function of spinal cord (SC) neural progenitor cells (NPCs).

**Methods:**

To identify potential therapeutic targets for these tumors, we leveraged primary mouse *Nf2*-deficient spinal cord neural progenitor cells.

**Results:**

We demonstrate that the *Nf2* protein, merlin, negatively regulates spinal neural progenitor cell survival and glial differentiation in an ErbB2-dependent manner, and that NF2-associated spinal ependymomas exhibit increased ErbB2 activation. Moreover, we show that *Nf2*-deficient SC NPC ErbB2 activation results from Rac1-mediated ErbB2 retention at the plasma membrane.

**Significance:**

Collectively, these findings establish ErbB2 as a potential rational therapeutic target for NF2-associated spinal ependymoma.

## Introduction

Spinal cord (SC) ependymomas are well-delineated gliomas characterized by prominent glial fibrillary acidic protein (GFAP) expression [1]. Converging genomic and cell biology evidence supports a radial glial/neural progenitor cell of origin for these tumors [2], [3]. As such, increasing neural progenitor cell (NPC) marker (nestin) expression has been associated with worse clinical outcome in patients with ependymoma [4]. Although ependymomas from different regions of the central nervous system (CNS) are histologically similar, they are clinically [5] and genetically [3], [6] distinct, suggesting that they represent a collection of different diseases with unique genetic drivers and responses to therapy. Currently, there are limited therapeutic options for ependymomas arising in the spinal cord, underscoring the need to identify the key molecular changes that specifically drive SC ependymoma growth in order to design treatments that target the signaling pathways de-regulated in these tumors.

Potential insights into the molecular pathogenesis of ependymoma are likely to derive from studies focused on familial syndromes in which affected individuals are prone to these tumors. One such syndrome, neurofibromatosis type 2 (NF2) is an autosomal dominant inherited cancer predisposition syndrome, caused by a germline mutation in the *NF2* tumor suppressor gene: 35–53% of individuals with NF2 develop ependymoma [7], with a striking predilection for the cervical or thoracic spinal cord (62–86% of tumors) [8]. The importance of the *NF2* gene to ependymoma pathogenesis is further emphasized by the observation that *NF2* gene mutation and loss of merlin expression is found in one-third of sporadic (non-syndromal) ependymomas [9]–[11]. Moreover, the majority of ependymomas exhibiting *NF2* loss occur in the spinal cord [12]. For these reasons, NF2 represents a tractable genetic model system to identify potential therapeutic targets for spinal ependymoma.

In the current study, we leveraged complementary genetic and pharmacologic approaches to demonstrate that merlin functions as a critical negative regulator of SC NPC survival and glial differentiation. Moreover, merlin controls NPC homeostasis by selectively suppressing ErbB2 activation, supported by the fact that both *Nf2*-deficient mouse SC NPCs and human NF2-patient spinal ependymomas exhibit increased ErbB2 activity. We further show that ErbB2 activation in *Nf2*-deficient SC NPCs requires Rac1 to retain ErbB2 in an active form at the plasma membrane. Because blockage of ErbB2 function reverses the enhanced survival and gliogenesis in *Nf2*-deficient SC NPCs, these studies establish ErbB2 as a potential rational therapeutic target for spinal ependymoma.

## Materials and Methods

### Ethics Statement

All mice were maintained in strict accordance with recommendations in the Guide for the Care and Use of Laboratory Animals of the National Institutes of Health and active animal studies protocols approved by the Animal Studies Committee at the Washington University School of Medicine (Protocol #20110111). All surgery was performed under isoflurane anesthesia, and all efforts were made to minimize suffering. All efforts were made to optimize experiments and reduce the number of animals used.

Human spinal cord and ependymoma sections were obtained from St. Jude Children’s Research Hospital (Memphis TN) and the Washington University School of Medicine Tissue Procurement Core (http://pathology.wustl.edu/research/cores/ltp/request.php), and used under an Institutional Review Board approved Human Studies Protocol (#201103323) to comply with ethical standards as well as government and institutional regulations. No direct patient consent was necessary, as these specimens were de-identified.

### Mice

*Nf2* ^flox/flox^ mice carrying a LoxP insertion in the *Nf2* gene [13] were maintained at the Washington University School of Medicine. Male and female embryos (E12.5) from mice maintained on a mixed C57BL/6 and FVB/N background were used for all experiments.

### Culture and Generation of *Nf2*−/− Neurospheres

Spinal cords dissected from E12.5 mouse *Nf2* ^flox/flox^ embryos were used to generate NPCs as previously reported [14]. Briefly, spinal cords were removed from embryonic day 12.5 (E12.5) decidua of timed-pregnant females and processed to obtain single-cell suspension of neural progenitors (neurospheres). Dissected spinal cords were digested with trypsin digest buffer containing 0.2% BSA (Sigma, St. Louis, MO), 0.5 mg/ml DNase I (Sigma), and 10% trypsin-EDTA stock (BioWhittaker, Walkersville, MD) in HBSS at 37°C for 15 min in a volume of 0.7 ml per litter. Equal volumes of 10% FBS medium containing 10% FBS (Life Technologies, Gaithersburg, MD) and low glucose DMEM/(Sigma) were added, and spinal cords were triturated with fire-polished Pasteur pipettes. Pelleted cells were washed with dissociation medium containing 15 mM HEPES (Sigma), 0.5% glucose in HBSS. Cells were finally resuspended in NSC medium containing a 5∶3 mixture of DMEM low glucose: Neurobasal medium (Life Technologies), 0.5 mM 2-mercaptoethanol, 2 mM L-glutamine, 5 IU of penicillin, and 5 µg/ml streptomycin (BioWhittaker) supplemented with 1% N2 supplement (Life Technologies), 2% B27 supplement (Life Technologies), 20 ng/ml epidermal growth factor (EGF) (Sigma), and 20 ng/ml basic fibroblast growth factor (FGF) (R & D Systems, Minneapolis, MN). Following infection with adenovirus containing either LacZ (wild-type (WT) NPCs) or Cre (*Nf2*−/− NPCs), merlin expression was determined by Western blotting (6 days after adenovirus infection).

### NPC Assays

Neurospheres were trypsinized (as described above) and plated into individual wells of ultralow-binding 24-well plates with complete NPC medium containing EGF and FGF. For assays in which the diameter and average number of neurospheres were measured, single neurospheres from WT and *Nf2−/−* NPCs were selected, trypsinized and individually plated into a single well of a 24-well plate. After 6 days, the size (diameter/cell growth) and number (clonogenic expansion) of the resulting secondary neurospheres was determined using Metamorph analysis software (Molecular Devices, Sunnyvale CA). Six wells per group per experiment were counted for these studies. For the proliferation assays, neurospheres from each group were trypsinized, 10^4^ cells were seeded in triplicate, and the number of cells assessed by direct cell counting (at 4, 6, and 8 days). Three wells per group per experiment were counted for these studies. Apoptosis was determined by counting the percent of cleaved caspase-3^+^ cells (1∶500 dilution; Cell Signaling Technology, Beverly MA) or Terminal deoxynucleotidyltransferase-mediated dUTP-biotin nick end labeling (TUNEL; Roche, Indianapolis IN)-immunoreactive cells relative to the total number of DAPI (4′, 6′-diamidino-2-phenylindole)-positive cells. Pharmacologic inhibitor experiments used 10^4^ cells per well (NSC23766, 10 µM; AG825, 10 µM; Sigma, St. Louis MO) for 6 days. All experiments were repeated at least three times with similar results.

### Viral Transduction

Murine stem cell virus (MSCV) infection was employed to express human WT or mutant (L64P patient mutation) merlin, mutant ErbB2 (V659E; Martine Roussel, St. Jude Children’s Research Hospital, Memphis TN), or dominant-negative Rac1 (Rac1^N17^) as previously described [15]. Mouse *Erbb2* (GenBank accession numbers NM_001003817.1 and XM_109715.3-689) small hairpin RNAs (shRNAs) lentiviral plasmids were obtained from the Washington University Genome Institute and generated as previously reported [16]. PLKO virus was used as an empty vector control. Viruses used are listed in Table S1. Neurospheres were trypsinized and plated with complete NSC media (as described above) in ultralow-attachment 100 mm dishes (Corning, NY) and virus was added (MOI-10) at 37°C overnight. Cells were then pelleted, plated in fresh complete NSC media and allowed to grow for 6 days. Neurospheres were then trypsinized and infected with adenovirus (as described above), and allowed to grow for 6 days. Cells were then trypsinized and assays were established accordingly.

### Western Blotting

Western blots were performed as previously described [17]. Briefly, neurospheres (after 6 days in culture) were lysed in MAPK lysis buffer (20 mM Tris (pH 7.0), 10 mM EGTA, 400 mM B-glycerophosphate, 1% NP-40, 2.5 mM MgCl_2_, and 2 mM sodium orthovanadate with protease inhibitors (leupeptin, aprotinin, and phenylmethylsulfonyl fluoride). For all experiments, 30 µg of protein per sample were loaded in each lane. Proteins were separated by SDS-polyacrylamide gel electrophoresis (SDS-PAGE) and transferred to polyvinylidene difluoride membranes (Millipore, Billerica, MA) prior to detection with phospho-specific antibodies. Antibodies that detect the total expression of the corresponding signaling intermediate as well as α-tubulin were used as controls for equal protein loading and quantitation. Active Rac1 (Rac1-GTP) was determined by PAK1-PBD affinity chromatography (Rac1 activation Assay Kit, Millipore, Bedford MA) according to the manufacturer’s recommendations. Appropriate HRP-conjugated secondary antibodies (Cell Signaling, Beverly MA) were used for detection by enhanced chemiluminescence (New England Biolabs, Beverly MA). Antibodies used are listed in Table S2.

### Subcellular Fractionation

Subcellular fractionation was performed as previously described [18]. Briefly, following hypotonic (10 mM HEPES at pH 7.9, 1.5 mM MgCl_2_, 10 mM KCl, protease inhibitors) lysis, nuclei were pelleted by centrifugation, and supernatants were subjected to ultracentrifugation at 100,000×g for 1 h at 4°C to generate cytosolic and membrane fractions. Equal percentages of each fraction (10 µl per lane (200 µl of total volume)) were subjected to SDS-PAGE and immunoblot analysis.

### Immunohistochemistry and Immunocytochemistry

Sections were treated with citrate antigen retrieval solution (10 mM, pH 6.0) and stained with pErbB2 (1∶50 dilution) as previously described [14]. Images were acquired on a Nikon Eclipse E600 microscope (Nikon Corporation, Tokyo, Japan) equipped with a Leica EC3 optical camera and Leica Application Suite 2.10 (Leica Microsystems, Wetzlar, Germany). Immunocytochemistry was performed on trypsinized NPCs grown in 50 µg/mL poly-D-lysine-coated and 10 µg/mL fibronectin-coated 24-well plates containing defined medium (5∶3 mixture of DMEM low glucose (Life Technologies): neurobasal medium (Life Technologies), 0.5 mM 2-mercaptoethanol, 2 mM L-glutamine, 5 IU penicillin, and 5 µg/ml streptomycin (Life Technologies) supplemented with 1% N2 supplement (Life Technologies) and 2% B27 supplement (Life Technologies)) without growth factors. After 5 days in culture, cells were fixed and stained with appropriate primary antibodies at 4°C overnight. For fluorescence detection, Alexa Fluor-tagged secondary antibodies (Molecular Probes, Eugene OR) were used and cells were counterstained with DAPI.

### Statistical Analysis

Statistical significance (p<0.05) was determined using the appropriate test as described in figure legends using GraphPad Prism 5.0 software (GraphPad Inc., La Jolla CA).

## Results and Discussion

### Merlin Negatively Regulates SC NPC Growth and Glial Differentiation

**Figure 1:**
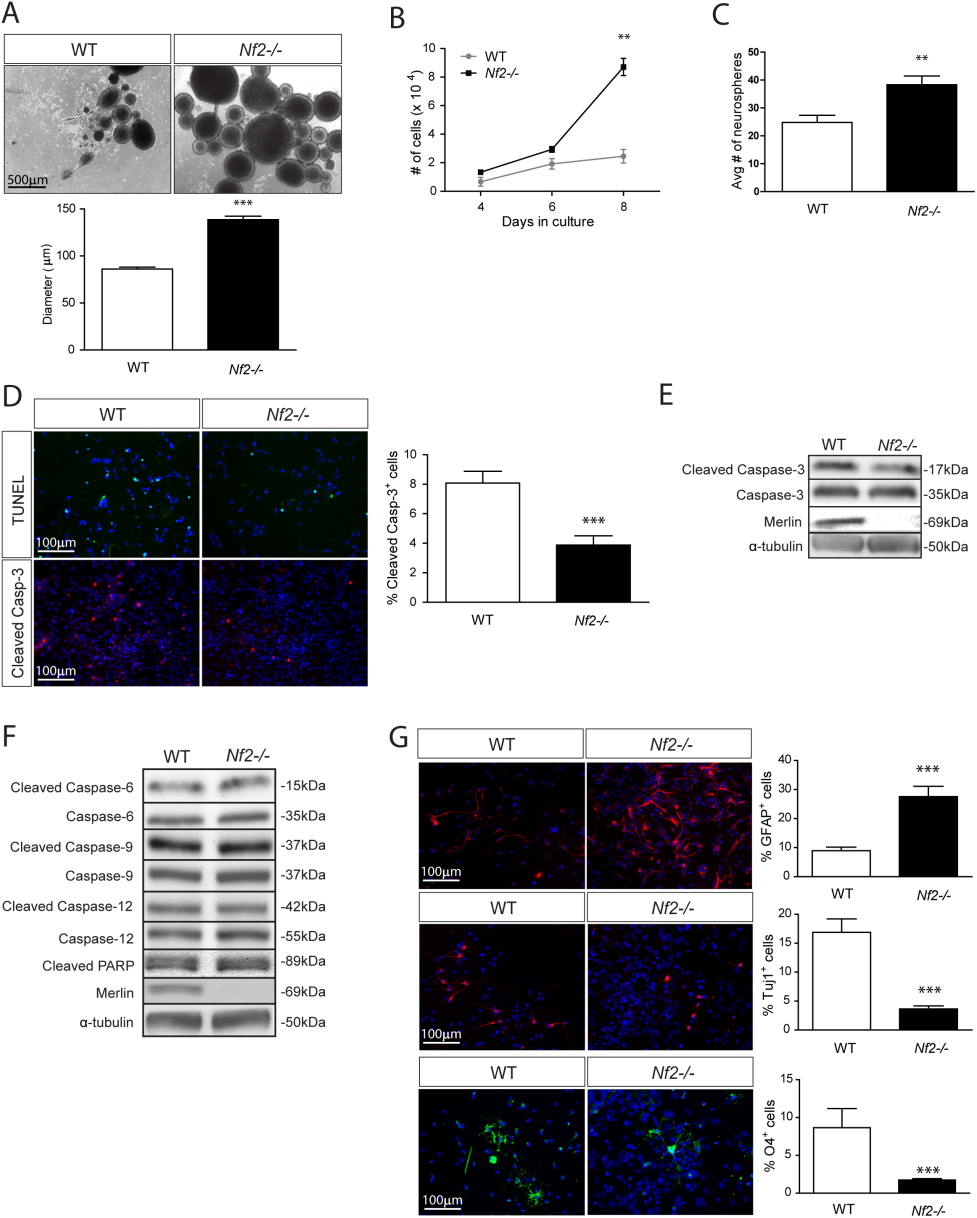
Merlin loss increases SC NPC growth, glial differentiation and cell survival. *Nf2* loss results in a (**A**) 1.8-fold increase in neurosphere diameter (p<0.0001; two-tailed Mann-Whitney U-test), (**B**) 3.6-fold increase in cell number at day 8 (p<0.001; 2-way ANOVA), and (**C**) a 1.7-fold increase in clonogenic expansion relative to WT SC NPCs (p = 0.003; two-tailed; two-tailed Mann-Whitney U-test). (**D, E**) *Nf2* loss results in a 2-fold decrease in cell death, as assessed by cleaved caspase-3 and TUNEL (p = 0.0008 and p = 0.0442; two-tailed Mann-Whitney U-test). (**F**) *Nf2* loss has no effect on the activation (cleavage) of caspase-6, -9, or -12 or PARP (statistics reported in Supplementary Figure 1C–F). (**G**) *Nf2 loss* results in increased glial (3-fold) (p = 0.0002; two-tailed Mann-Whitney U-test), but decreased neuronal (4.6-fold) (p<0.0001; two-tailed Mann-Whitney U-test) and oligodendrocyte (5-fold) differentiation (p<0.0001; two-tailed Mann-Whitney U-test). The data were normalized to the field of view. Values denote the mean ± SEM. (*) p<0.05; (**) p<0.001; (***) p<0.0001.

Since spinal cord ependymomas are hypothesized to originate from radial glial-like stem/progenitor cells [2], [3] expressing the fatty acid binding protein-7 (brain lipid binding protein, BLBP) (Fig. S1A), initial studies employed genetically-engineered mice in which the *NF2* gene was conditionally deleted in BLBP^+^ cells (*Nf2* ^BLBP^CKO mice) using a previously published BLBP-Cre strain [19]. However, no viable *Nf2* ^BLBP^CKO pups were born, likely due to a requirement for merlin expression in BLBP^+^ cells during mid-embryonic development (E9.5) [19]. For this reason, we chose to employ primary embryonic spinal cord (SC) NPC cultures from *Nf2* floxed mice (*Nf2^flox/flox^* ^;^ [13]) in which merlin expression could be eliminated following adenoviral delivery of Cre recombinase (*Ad5Cre*). Consistent with an essential role for merlin in SC NPC homeostasis, *Nf2* inactivation results in increased NPC growth (1.8-fold increase in neurosphere diameter, Fig. 1A; 3.6-fold increase in cell number at day 8, Fig. 1B) and a 1.7-fold increase in clonogenic expansion (secondary neurosphere formation) (Fig. 1C). To define the cellular mechanism responsible for merlin regulation of NPC growth, we initially measured programmed cell death. Following *Nf2* inactivation, there was a 2-fold decrease in apoptosis as measured by cleaved caspase-3 immunocytochemistry and Western blotting (Fig. 1D and E) as well as by TUNEL staining (Fig. 1D). No changes in the activity (cleavage) of other caspase family members were observed in *Nf2*-deficient SC NPCs (Fig. 1F and Fig. S1B–F).

Since ependymomas are glial cell tumors, we next sought to determine whether *Nf2* loss in NPCs increases gliogenesis. Following *in vitro* differentiation, merlin loss results in a 3-fold increase in glial differentiation relative to *Ad5LacZ*-infected (wild-type; WT) SC NPCs. Following merlin loss, there is also a decrease in neuronal (4.7-fold decrease) and oligodendroglial (5-fold decrease) differentiation (Fig. 1G).

**Figure 2:**
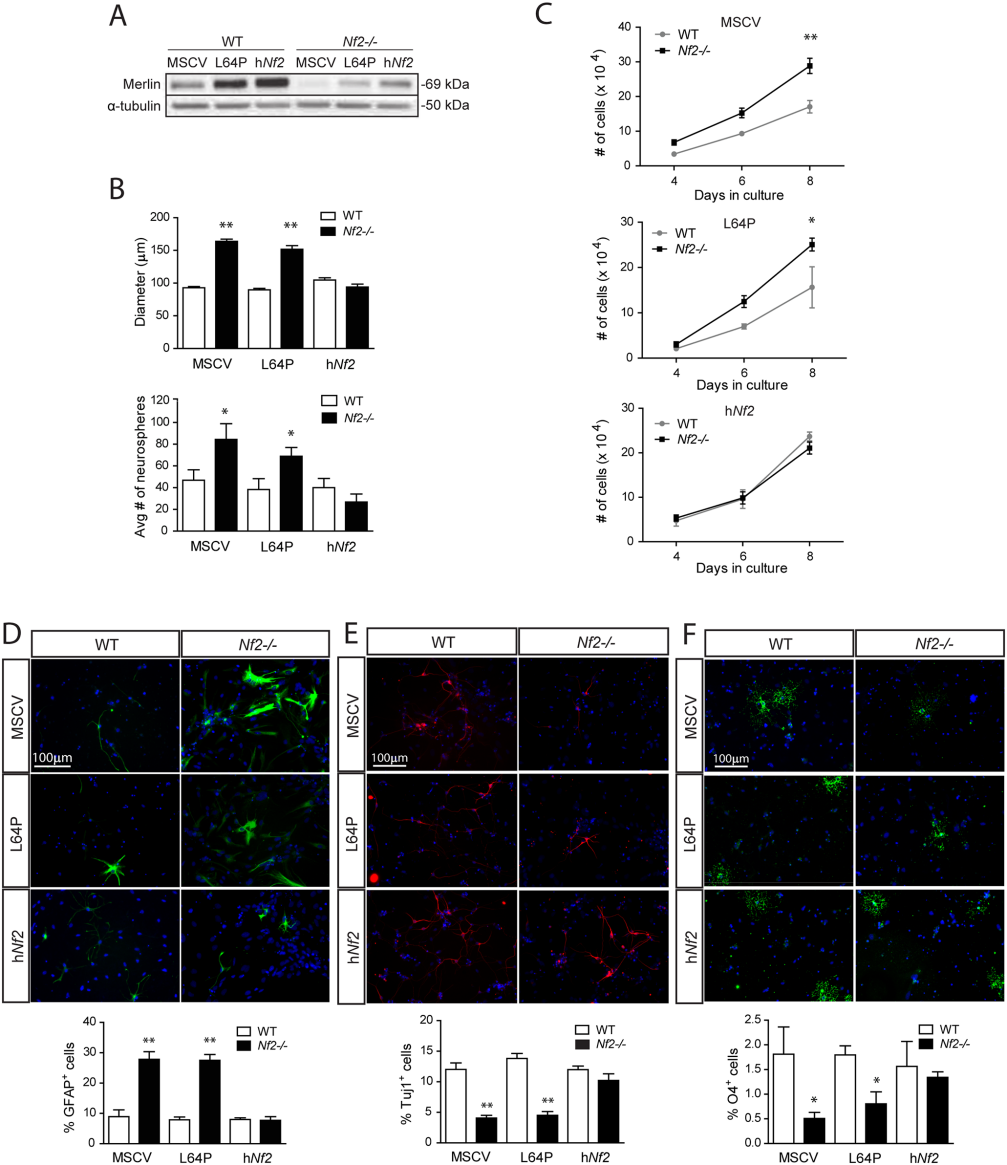
Wild-type, but not mutant, merlin re-expression restores SC NPC properties to WT levels. (**A**) Re-expression of WT (MSCV.NF2), but not mutant (MSCV.NF2.L64P), merlin reduced (**B**) SC NPC growth (diameter) and clonogenic expansion (neurospheres) to WT levels (diameters: MSCV, p<0.001; L64P, p<0.001; h*Nf2*, p = 0.1584; average number of neurospheres: MSCV, p<0.0421; L64P, p<0.05; h*Nf2*, p = 0.7138; two-way ANOVA with Bonferroni post-test). (**C**) Wild-type (WT; MSCV.*NF2*), but not mutant (MSCV.*NF2*.L64P), merlin expression in *Nf2−/−* SC NPCs decreases cell number at day 8 (MSCV, p<0.001; L64P, p<0.05; h*Nf2*, p = 0.200; two-way ANOVA with Bonferroni post-test) and (**D**) glial differentiation to WT levels (MSCV, p<0.001; L64P, p<0.001; h*Nf2*, p = 0.7238; two-way ANOVA with Bonferroni post-test). Wild-type (WT; MSCV.*NF2*), but not mutant (MSCV.*NF2*.L64P), merlin expression in *Nf2−/−* NPCs increases (**E**) neuronal (MSCV, p<0.001; L64P, p<0.001; h*Nf2*- p = 0.1797; two-way ANOVA with Bonferroni post-test) and (**F**) oligodendrocyte differentiation to WT levels (MSCV, p<0.01; L64P, p<0.01; h*Nf2*, p = 0.7308; two-way ANOVA with Bonferroni post-test. The data were normalized to the field of view. Values denote the mean ± SEM. (*) p<0.05; (**) p<0.001; (***) p<0.0001.

To establish a causal relationship between merlin loss and SC NPC function, merlin was re-expressed in *Nf2*-deficient NPCs by retroviral infection (Fig. 2A). In these experiments, expression of WT, but not NF2-patient mutant (L64P; [20], [21]), merlin restored SC NPC neurosphere diameters (Fig. 2B), clonogenic expansion (Fig. 2B), cell number (Fig. 2C), and multi-lineage differentiation (Fig. D–F) to WT levels. Collectively, these results demonstrate that merlin is a direct regulator of SC NPC growth and glial differentiation in vitro.

### Merlin Negatively Controls ErbB2 Activation

**Figure 3:**
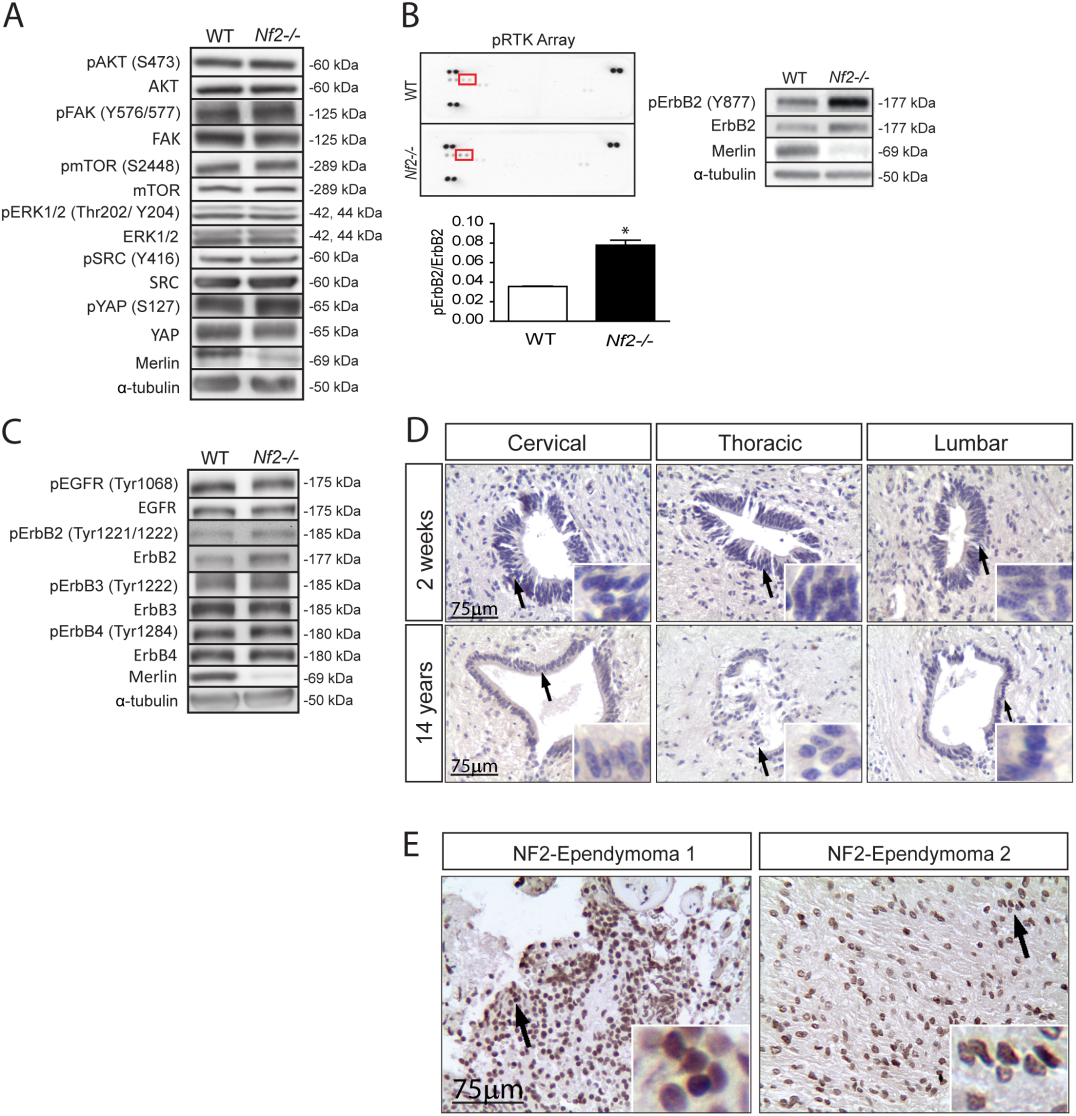
Merlin loss leads to increased ErbB2 activation. (**A**) *Nf2* loss does not change the activation status of AKT (S473), FAK (Y576/577), mTOR (S2448), ERK1/2 (Thr202/Y204), SRC (Y416), or YAP (S127). The quantification of all independent experiments is shown in Fig. S2A–I. (**B**) *Nf2* loss results in a 2-fold increase in ErbB2 hyperactivation initially demonstrated with a commercial activated RTK array (left panel) and subsequently confirmed using independent samples and a phospho-specific ErbB2 antibody (Tyr^877^ phosphorylation; bottom and right panel) (p = 0.0145; two-tailed Mann-Whitney U-test). (**C**) *Nf2* loss does not increase the phosphorylation (activation) status of EGFR (Tyr1068), ErbB2 (Tyr1221/1222), ErbB3 (Tyr1222), or ErbB4 (Tyr1284). The quantification of all independent experiments is shown in Fig. S2J–M. (**D**) Normal human spinal cords lack ErbB2 activation, whereas (**E**) robust pErbB2 immunoreactivity was observed in two representative NF2-patient ependymomas. Values denote the mean ± SEM. (*) p<0.05; (**) p<0.001; (***) p<0.0001.

To define the mechanism underlying merlin regulation of NPC homeostasis, we examined the activation status of signaling pathways previously shown to govern *Nf2*-deficient cell growth, including AKT, FAK, mTOR, YAP, and SRC [17], [22]–[24]. However, none of these signaling intermediates/pathways were hyperactivated following *Nf2* loss in SC NPCs (Fig. 3A and Fig. S2A–I). Next, we sought to determine whether merlin growth regulation involves suppression of receptor tyrosine kinase (RTK) activation, based on previous studies in differentiated forebrain astrocytes [17] and other non-nervous system cell types [25]. Using a commercial activated RTK array, the only RTK in *Nf2*-deficient SC NPCs with significant activation over their WT counterparts was ErbB2 (Fig. 3B, top left panel). We confirmed this result using independent *Nf2*−/− SC NPC cultures, and found a 2-fold increase in ErbB2 activation relative to WT SC NPCs using Y877 phospho-specific antibodies (Fig. 3B, bottom and right panel). Hyperactivation of other ErbB family members (EGFR, ErbB3, ErbB4) using phospho-specific antibodies was not observed (Fig. 3C and Fig. S2J–M).

Since over 75% of pediatric ependymomas express ErbB2 [26] and increased ErbB family receptor tyrosine kinase signaling has been reported in the hallmark NF2-associated tumor (vestibular schwannoma) [27], [28] we examined ErbB2 activation in normal human spinal cord and in two representative spinal ependymomas from individuals with a confirmed diagnosis of NF2. These tumors are not typically removed, thus limiting a more exhaustive analysis. While normal spinal cord autopsy specimens from individuals ranging from 2 weeks to 14 years of age lacked ErbB2 activation (Fig. 3D), the two human NF2-patient ependymoma samples exhibited robust phospho-ErbB2 expression (Fig. 3E).

### Merlin Regulates SC NPC Growth and Glial Differentiation in an ErbB2-dependent Manner

**Figure 4:**
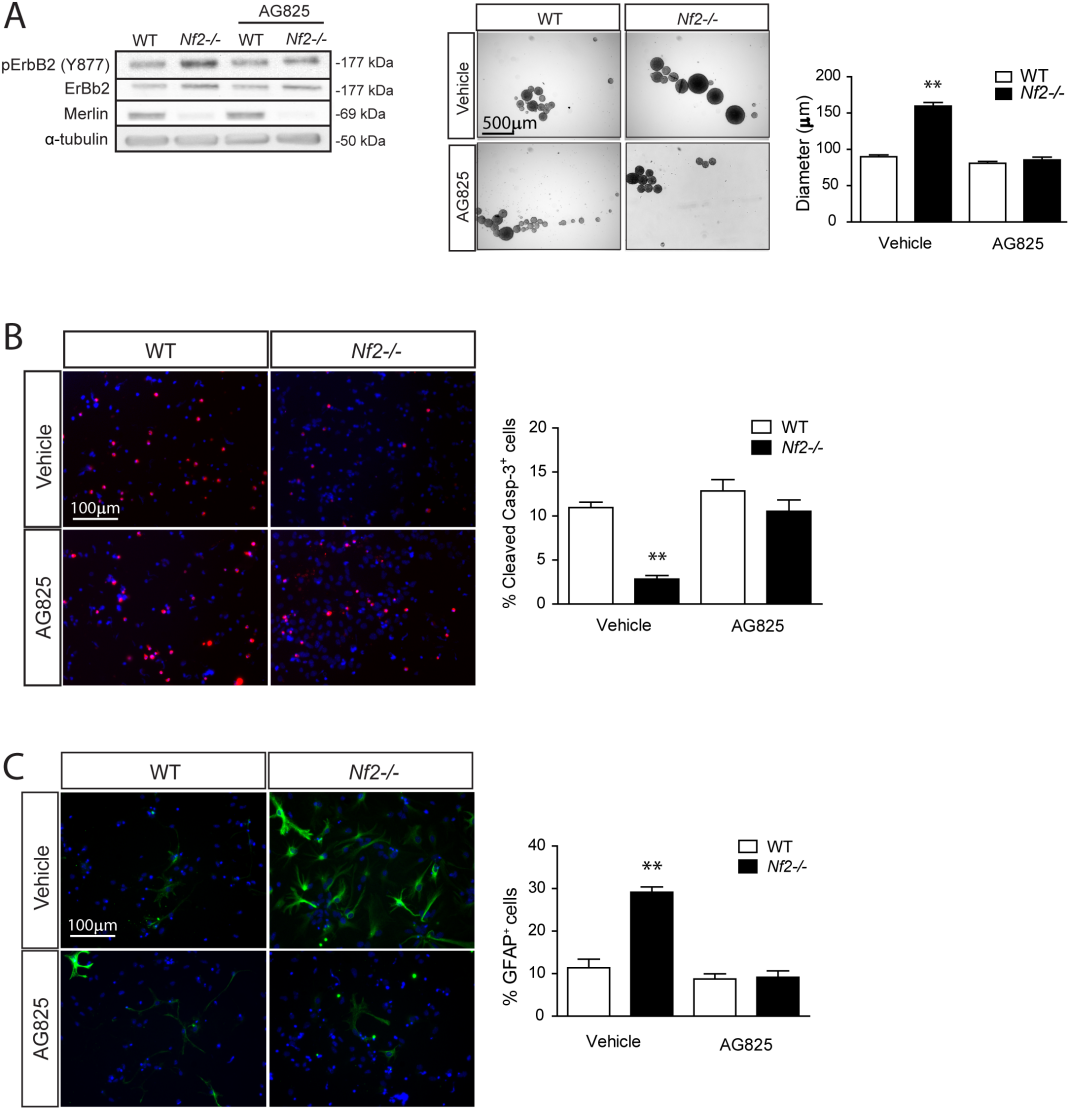
Increased *Nf2*-deficient SC NPC growth and glial differentiation is reversed by pharmacologic ErbB2 inhibition. (**A**) Pharmacologic ErbB2 inhibition (AG825; 10 µM) reduces *Nf2−/−* SC NPC neurosphere diameters (Vehicle: WT vs. *Nf2−/−,* p<0.001; AG825: WT vs. *Nf2−/−*, p = 0.2308; two-way ANOVA with Bonferroni post-test) and (**B**) increases cell death (% cleaved caspase-3^+^ cells) to WT levels (Vehicle: WT vs *Nf2−/−*, p<0.001; AG825: WT vs. *Nf2−/−*, p = 0.1903; two-way ANOVA with Bonferroni post-test). (**C**) Pharmacologic ErbB2 inhibition (AG825; 10 µM) reduces *Nf2−/−* SC NPC glial differentiation to WT levels (Vehicle: WT vs. *Nf2−/−*, p<0.001; AG825: WT vs. *Nf2−/−*, p = 0.8633; two-way ANOVA with Bonferroni post-test). The data were normalized to the field of view. Values denote the mean ± SEM. (*) p<0.05; (**) p<0.001; (***) p<0.0001.

**Figure 5:**
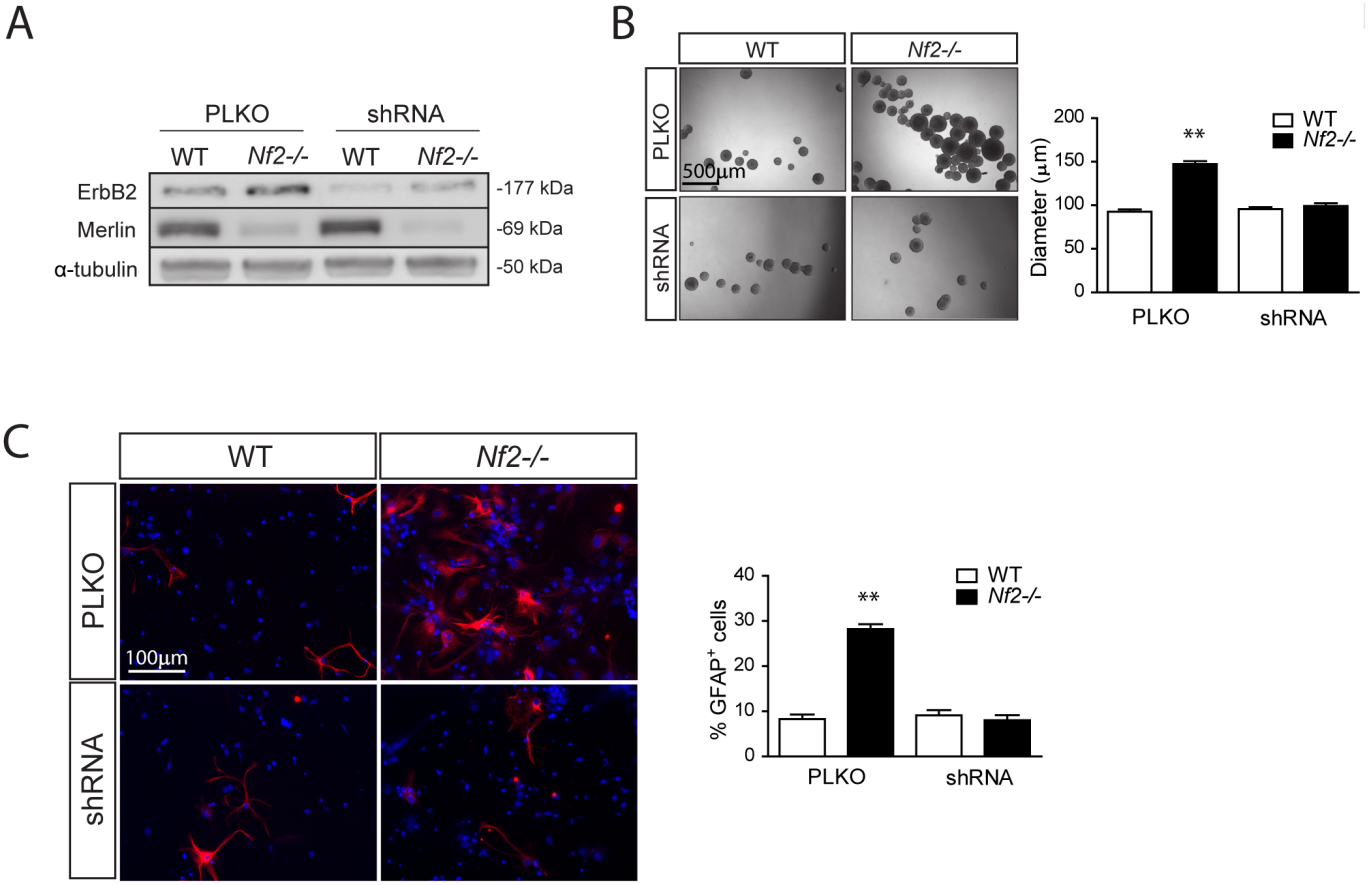
Increased *Nf2*-deficient SC NPC growth and glial differentiation is reversed by genetic ErbB2 silencing. (**A**) shRNA-mediated ErbB2 silencing (**B**) decreases *Nf2−/−* SC NPC neurosphere diameters (PLKO: WT vs. *Nf2−/−*, p<0.001; shRNA: WT vs. *Nf2−/−*, p = 0.3821; two-way ANOVA with Bonferroni post-test) (**C**) and glial differentiation to WT levels (PLKO: WT vs. *Nf2−/−*, p<0.001. shRNA: WT vs. *Nf2−/−*, p = 0.3704; two-way ANOVA with Bonferroni post-test). The data were normalized to the field of view. Values denote the mean ± SEM. (*) p<0.05; (**) p<0.001; (***) p<0.0001.

To establish an essential role for ErbB2 activation in merlin regulation of SC NPC function, we employed complementary pharmacological and genetic approaches. In these studies, pharmacologic ErbB2 inhibition (tyrphostin; AG825) decreased *Nf2*−/− NPC neurosphere diameters (Fig. 4A), cell survival (Fig. 4B), and glial differentiation (Fig. 4C) to WT levels. Similar results were also obtained using another pharmacological ErbB2 inhibitor (lapatinib; Fig. S2N). Moreover, shRNAi silencing of ErbB2 expression using two different *Erbb2* shRNA constructs (Fig. 5A) reduced *Nf2*−/− SC NPC neurosphere diameters (Fig. 5B) and glial differentiation (Fig. 5C) to WT levels. While we favor the hypothesis that merlin regulates SC NPC cell survival and glial differentiation in an ErbB2-dependent manner, these two processes do not have to be linked and may involve separate downstream signaling pathways.

**Figure 6:**
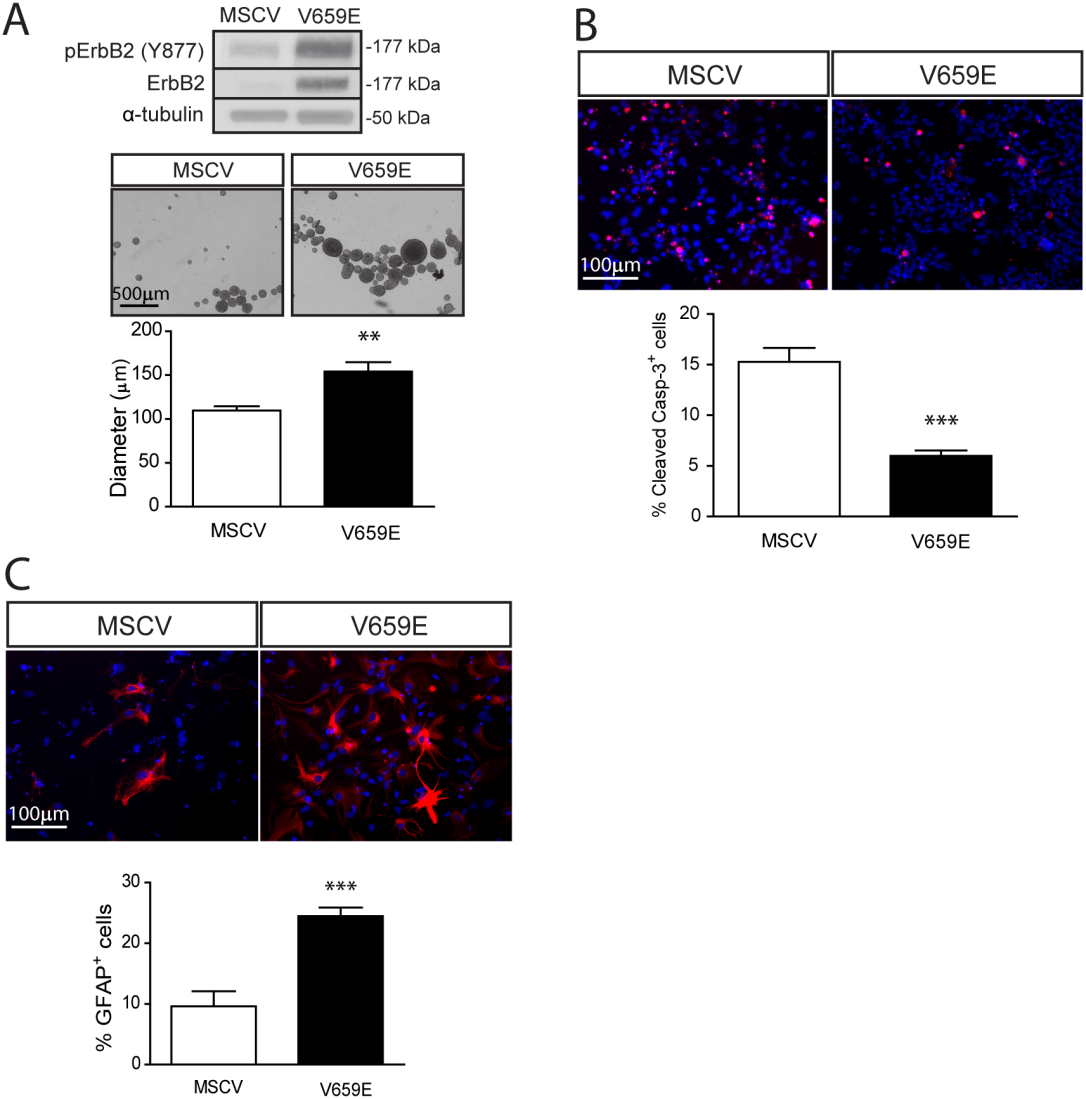
Expression of an activated ErbB2 molecule mimics *Nf2* loss in SC NPCs. (**A**) Constitutively-activated ErbB2 (V659E) expression increases SC NPC neurosphere diameters (1.4-fold) (p = 0.0021; two-tailed Mann-Whitney U-test), (**B**) decreases NPC apoptosis (% cleaved caspase-3^+^ cells; 2.6-fold) (p<0.0001; two-tailed Mann-Whitney U-test), and (**C**) increases glial differentiation (2.6-fold) (p = 0.0006; two-tailed Mann-Whitney U-test). The data were normalized to the field of view. Values denote the mean ± SEM. (*) p<0.05; (**) p<0.001; (***) p<0.0001.

To further support ErbB2 activation as an important regulator of SC NPC homeostasis, we overexpressed a constitutively-activated ErbB2 (V659E) molecule by MSCV retroviral infection. Expression of ErbB2-V659E in WT SC NPCs resulted in a 1.4-fold increase in cell growth (neurosphere diameter; Fig. 6A), a 2.6-fold decrease in cell death (Fig. 6B), and a 2.6-fold increase in glial differentiation (Fig. 6C) relative to vector (MSCV) controls. Collectively, these results establish that ErbB2 is both necessary and sufficient for merlin regulation of SC NPC growth and glial differentiation.

### Merlin Controls SC NPC Function by Regulating ErbB2 Plasma Membrane Localization and Signaling in a Rac1-dependent Manner

**Figure 7:**
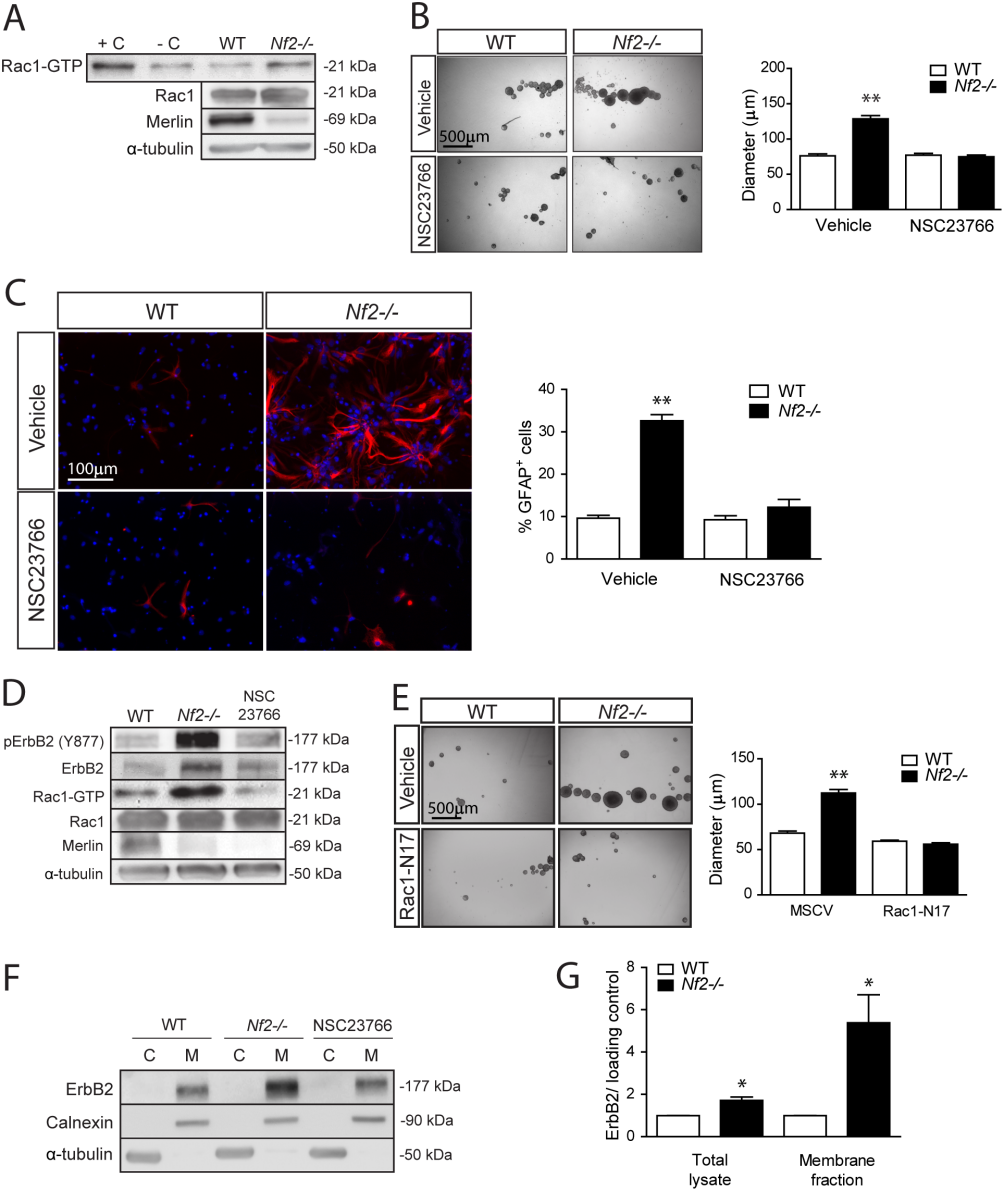
Merlin negatively regulates SC NPC growth and glial differentiation in a Rac1- and ErbB2-dependent manner. (**A**) *Nf2* loss results in a 2.6-fold increase in Rac1 activity (statistical analysis shown in Supplementary Figure 2O). (**B**) Pharmacologic Rac1inhibition decreases *Nf2*−/− SC NPC neurosphere diameters (Vehicle: WT vs. *Nf2−/−*  = , p<0.001; NSC23766: WT vs. *Nf2−/−*, p = 0.9540; two-way ANOVA with Bonferroni post-test) (**C**) and glial differentiation to WT levels. (Vehicle: WT vs. *Nf2−/−*, p<0.001; NSC23766: WT vs. *Nf2−/−*, p = 0.1738; two-way ANOVA with Bonferroni post-test). (**D**) Pharmacologic Rac1 inhibition decreases *Nf2−/−* SC NPC ErbB2 hyperactivation. (**E**) Genetic Rac1 inhibition (Rac1^N17^ expression) reduces *Nf2−/−* NPC neurosphere diameters to WT levels. (MSCV: WT vs. *Nf2−/−*, p<0.001; Rac1^N17^: WT vs. *Nf2−/−*, p = 0.0263; two-way ANOVA with Bonferroni post-test). (**F**) *Nf2−/−* SC NPCs exhibit a 5.4-fold enrichment of ErbB2 in the plasma membrane fraction (p = 0.0265; two tailed Mann-Whitney U-test). Rac1 inhibition (NSC23766) decreased plasma membrane ErbB2 expression to WT levels. (**G**) *Nf2−/−* SC NPCs have a 2.7-fold increase in ErbB2 enrichment in the membrane fraction when normalized to total ErbB2 levels in the total cell lysates (2.0-fold increase) (p<0.05; two-way ANOVA with Bonferroni post-test). The data were normalized to the field of view. Values denote the mean ± SEM. (*) p<0.05; (**) p<0.001; (***) p<0.0001.

Next, we sought to define the mechanism underlying merlin regulation of ErbB2 activation. Western blot analysis revealed that both phosphorylated and total ErbB2 protein levels were increased in *Nf2*-deficient SC NPCs, suggesting that the observed de-regulated ErbB2 signaling could result from increased plasma membrane localization. Previous studies from our laboratory and others have implicated merlin in cytoskeleton dynamics [29], [30] raising the intriguing possibility that ErbB2 membrane localization and signaling is controlled by Rac1, a downstream merlin target important for actin cytoskeleton function [31]–[34]. Consistent with this model, Rac1 activation was increased 2.6-fold in *Nf2*−/− SC NPCs relative to their WT counterparts (Fig. 7A and Fig. S2O). Moreover, pharmacologic Rac1 inhibition (NSC23766) decreased *Nf2*-deficient NPC neurosphere diameters (Fig. 7B) and glial differentiation (Fig. 7C) to WT levels. To confirm that Rac1 is the effector responsible for ErbB2 hyperactivation in *Nf2*−/− SC NPCs, we found that pharmacologic Rac1 inhibition decreased ErbB2 activity (Fig. 7D). Similar results were also obtained using a dominant-negative Rac1 (Rac1^N17^) molecule (Fig. 7E).

Previous studies in non-nervous system cell types have shown that merlin loss increases the internalization of another RTK (EGFR) [35], [36]. To determine whether this mechanism might underlie merlin regulation of ErbB2 activation in *Nf2*-deficient SC NPCs, cell fractionation was performed. In these experiments, *Nf2*−/− SC NPCs exhibit a 2.7-fold enrichment of ErbB2 in the plasma membrane fraction (5.4-fold increase) when normalized to total ErbB2 levels in the total cell lysates (2.0-fold increase) (Fig. 7F, G). These results support a model in which ErBb2 localization to the membrane allows ErbB2 to remain in an active, hyper-phosphorylated state. To determine whether Rac1 controls ErbB2 plasma membrane enrichment, we demonstrate that Pharmacologic Rac1 inhibition (NSC23766) decreased plasma membrane ErbB2 expression to WT levels (Fig. 7F). Importantly, β-catenin (a known regulator of cell-cell adhesion) is not hyperactivated in *Nf2−/−* NPCs (Fig. S2P). Collectively, these findings establish that merlin negatively controls NPC function by regulating ErbB2 plasma membrane localization and signaling in a Rac1-dependent manner.

## Conclusions

Spinal cord ependymomas are non-malignant neoplasms with limited chemotherapeutic options. Currently, the only effective treatment for clinically-symptomatic tumors is surgery, which carries significant potential morbidity. In this report, we leveraged *Nf2−/−* SC NPCs as an in vitro model system to define the growth control mechanism underlying spinal ependymoma pathogenesis relevant to the implementation of biologically-targeted treatments for these tumors. The finding that merlin regulates SC NPC growth in a Rac1- and ErbB2-dependent manner firmly establishes a central growth control target for NF2-associated spinal ependymoma, and advocate for further studies addressing the potential use of ErbB2 inhibitors to treat these CNS neoplasms.

The importance of ErbB2 in NF2-associated tumors is also highlighted by recent research showing that lapatinib inhibits vestibular schwannoma growth [28]. Because ependymomas are thought to arise from radial glial-like progenitor cells in the spinal cord^3^, the important relationship between ErbB2 function and ependymoma is further underscored by the observation that this specific RTK is required for the establishment and maintenance of CNS radial glia [37], [38]. In addition, ErbB2 is one of the most frequently mutated genes in human high-grade glioma [39], such that ErbB2 activation promotes glioma and ependymoma cell line growth in vitro [26], [40]. Similar to our findings in *Nf2*-deficient SC NPCs, ErbB2 increases glioma cell growth by inhibiting apoptosis [40]. Taken together, our findings demonstrate that ErbB2 is a critical regulator of *Nf2*-deficient SC NPC survival and glial differentiation, and support further evaluation in preclinical and human clinical trials for NF2-associated and select sporadic ependymomas.

## Supporting Information

Figure S1 **Merlin re-expression restores** ***Nf2-*** **deficient SC NPC growth and differentiation to wild-type levels.** (**A**) The mouse SC ependymal cell layer is immunopositive for fatty acid binding protein-7 (brain lipid binding protein, BLBP) expression. (**B**) *Nf2-*deficient SC NPCs have a 2-fold decrease in apoptosis as measured by cleaved caspase-3 (p = 0.0442; two-tailed Mann-Whitney U-test). (**C–F**) No changes in the activity (cleavage) of other caspase family members were observed in *Nf2*-deficient SC NPCs (C. Casp-6 –p = 0.6579; C. Casp-9 –p = 0.6579; C. Casp-12 –p = 0.7000; C. PARP –p = 0.1840; two-tailed Mann-Whitney U-test). Values denote the mean ± SEM. (*) p<0.05; (**) p<0.001; (***) p<0.0001.
(TIF)

Figure S2 (**A–I**) *Nf2* loss does not lead to changes in the activation status of signaling pathways previously implicated in *Nf2*-deficient cell growth regulation, including AKT (S473, p = 0.8857; Thr308, p = 0.6579), FAK (Y576/577, p = 0.6579), mTOR (S2448, p = 0.6579; Y2446, p = 0.8251), ERK1/2 (Thr202/Y204, p = 0.3429), SRC (Y416, p = 0.8857), and YAP (S127, p = 0.6579) (two-tailed Mann-Whitney U-test). (**J–M**) Hyperactivation of other ErbB family members using phospho-specific antibodies was not observed (EGFR Tyr1068, p = 0.6579; ErbB2 Y1221–1222, p = 0.4; ErbB3 Tyr1222, p = 1.000; and ErbB4 Tyr1284, p = 0.9162) (two-tailed Mann-Whitney U-test). (**N**) Lapatinib decreases neurosphere diameters in *Nf2*-deficient SC NPCs (p<0.001; two-way ANOVA with Bonferroni post-test). (**O**) *Nf2-*deficient SC NPCs exhibit a 2.6-fold increase in Rac1 activity compared to WT SC NPCs (p = 0.0109); two-tailed Mann-Whitney U-test). (**P**) β-catenin (regulator of cell-cell adhesion) expression is not changed in *Nf2−/−* NPCs relative to their WT counterparts (p = 0.9600; two-tailed Mann-Whitney U-test). Values denote the mean ± SEM. (*) p<0.05; (**) p<0.001; (***) p<0.0001.
(TIF)

Table S1 **Plasmids.** Murine stem cell virus (MSCV) and lentiviral plasmids used.
(DOCX)

Table S2 **Antibodies.** Antibodies used, source and dilutions. Immunocytochemistry (ICC), Immunohistochemistry (IHC) and Western Blot (WB), Ms: Mouse antibody; Rb: Rabbit antibody, Cl. = cleaved, p- = phospho-.
(DOCX)
