# Supplementary figures and images for: Nf2/Merlin Controls Spinal Cord Neural Progenitor Function in a Rac1/ErbB2-Dependent Manner
Source: PLoS One. 2014 May 9;9(5):e97320. doi: 10.1371/journal.pone.0097320 (PMC4016309; doi:10.1371/journal.pone.0097320)

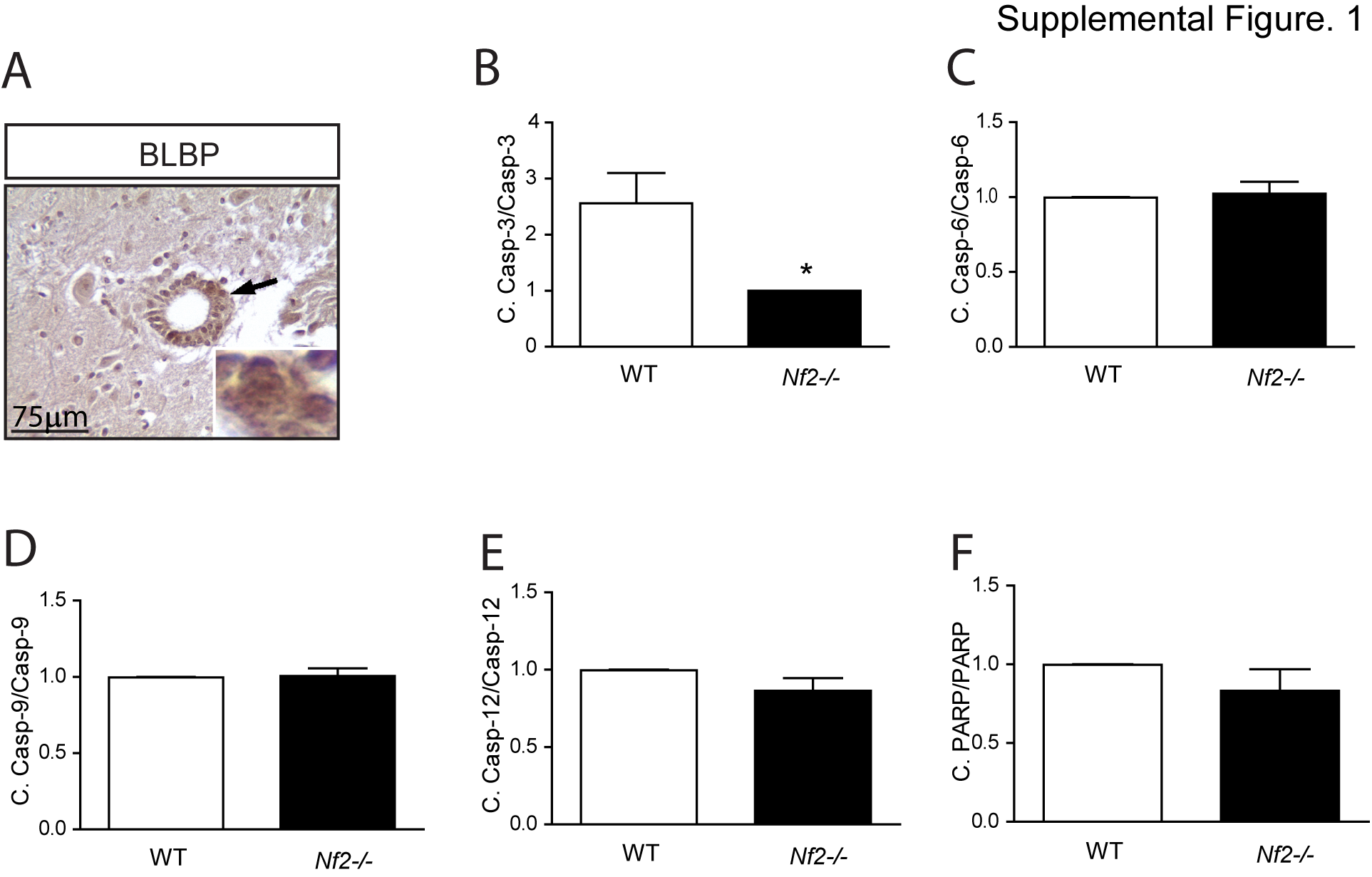

Supplement: Figure S1 — Merlin re-expression restores Nf2- deficient SC NPC growth and differentiation to wild-type levels. (A) The mouse SC ependymal cell layer is immunopositive for fatty acid binding protein-7 (brain lipid binding protein, BLBP) expression. (B) Nf2-deficient SC NPCs have a 2-fold decrease in apoptosis as measured by cleaved caspase-3 (p = 0.0442; two-tailed Mann-Whitney U-test). (C–F) No changes in the activity (cleavage) of other caspase family members were observed in Nf2-deficient SC NPCs (C. Casp-6 –p = 0.6579; C. Casp-9 –p = 0.6579; C. Casp-12 –p = 0.7000; C. PARP –p = 0.1840; two-tailed Mann-Whitney U-test). Values denote the mean ± SEM. (*) p<0.05; (**) p<0.001; (***) p<0.0001. (TIF) [file pone.0097320.s001.tif]

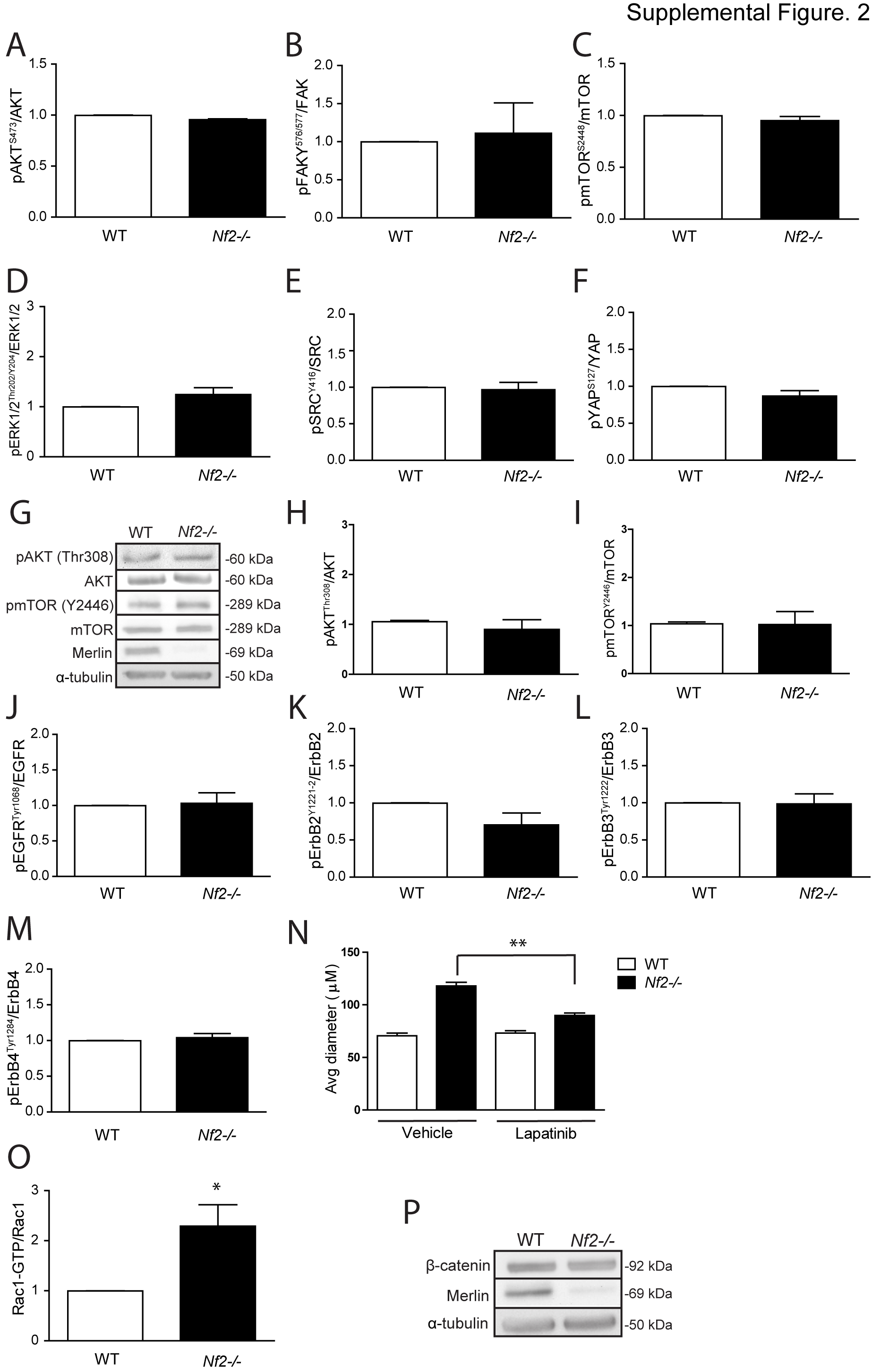

Supplement: Figure S2 — (A–I) Nf2 loss does not lead to changes in the activation status of signaling pathways previously implicated in Nf2-deficient cell growth regulation, including AKT (S473, p = 0.8857; Thr308, p = 0.6579), FAK (Y576/577, p = 0.6579), mTOR (S2448, p = 0.6579; Y2446, p = 0.8251), ERK1/2 (Thr202/Y204, p = 0.3429), SRC (Y416, p = 0.8857), and YAP (S127, p = 0.6579) (two-tailed Mann-Whitney U-test). (J–M) Hyperactivation of other ErbB family members using phospho-specific antibodies was not observed (EGFR Tyr1068, p = 0.6579; ErbB2 Y1221–1222, p = 0.4; ErbB3 Tyr1222, p = 1.000; and ErbB4 Tyr1284, p = 0.9162) (two-tailed Mann-Whitney U-test). (N) Lapatinib decreases neurosphere diameters in Nf2-deficient SC NPCs (p<0.001; two-way ANOVA with Bonferroni post-test). (O) Nf2-deficient SC NPCs exhibit a 2.6-fold increase in Rac1 activity compared to WT SC NPCs (p = 0.0109); two-tailed Mann-Whitney U-test). (P) β-catenin (regulator of cell-cell adhesion) expression is not changed in Nf2−/− NPCs relative to their WT counterparts (p = 0.9600; two-tailed Mann-Whitney U-test). Values denote the mean ± SEM. (*) p<0.05; (**) p<0.001; (***) p<0.0001. (TIF) [file pone.0097320.s002.tif]
