# Supplementary material for: Nf2/Merlin Controls Spinal Cord Neural Progenitor Function in a Rac1/ErbB2-Dependent Manner
Source: PLoS One. 2014 May 9;9(5):e97320. doi: 10.1371/journal.pone.0097320 (PMC4016309; doi:10.1371/journal.pone.0097320)
Supplement: Table S1 — Plasmids. Murine stem cell virus (MSCV) and lentiviral plasmids used. (DOCX) [file pone.0097320.s003.docx]

**Table S1. Plasmids.**

| **Construct** | **Source** |
| --- | --- |
| *ErbB2* shRNA; NM_001003817.1, XM_109715.3-689 | Washington University Genome Institute, St. Louis MO |
| pLKO.1 | Sigma, St. Louis MO |
| MSCV.ErbB2^V659E^ | Martine Roussel, St. Jude Children's Research Hospital, Memphis TN |
| MSCV.NF2 (L64P) | Houshmandi et al., 2009 |
| MSCV.NF2 (WT) | Houshmandi et al., 2009 |
| MSCV.Rac1^N17^ | Banerjee et al., 2010 |
