# Supplementary material for: Nf2/Merlin Controls Spinal Cord Neural Progenitor Function in a Rac1/ErbB2-Dependent Manner
Source: PLoS One. 2014 May 9;9(5):e97320. doi: 10.1371/journal.pone.0097320 (PMC4016309; doi:10.1371/journal.pone.0097320)
Supplement: Table S2 — Antibodies. Antibodies used, source and dilutions. Immunocytochemistry (ICC), Immunohistochemistry (IHC) and Western Blot (WB), Ms: Mouse antibody; Rb: Rabbit antibody, Cl. = cleaved, p- = phospho-. (DOCX) [file pone.0097320.s004.docx]

**Table S2. Antibodies.**

| **Antibody** | **Source** | **Dilution** |
| --- | --- | --- |
| AKT (WB); Rb | Cell Signaling (Beverly MA) | 1:1000 |
| BLBP (IHC); Rb | Chemicon (Temecula CA) | 1:2000 |
| Calnexin (WB); Rb | Enzo (Farmingdale NY) | 1:1000 |
| Caspase-3 (WB); Rb | Cell Signaling (Beverly MA) | 1:1000 |
| Caspase-6 (WB); Rb | Cell Signaling (Beverly MA) | 1:1000 |
| Caspase-9 (WB); Rb | Cell Signaling (Beverly MA) | 1:1000 |
| Caspase-12 (WB); Rb | Cell Signaling (Beverly MA) | 1:1000 |
| Cl. Caspase-3 (ICC; WB); Rb | Cell Signaling (Beverly MA) | 1:500 |
| Cl. Caspase-6 (WB); Rb | Cell Signaling (Beverly MA) | 1:1000 |
| Cl. Caspase-9 (WB); Rb | Cell Signaling (Beverly MA) | 1:1000 |
| Cl. Caspase-12 (WB); Rb | Cell Signaling (Beverly MA) | 1:1000 |
| Cl. PARP (WB); Rb | Cell Signaling (Beverly MA) | 1:1000 |
| EGFR (WB); Rb | Cell Signaling (Beverly MA) | 1:1000 |
| ErbB2 (ICC, WB); Rb | Abcam (Cambridge MA) | 1:100, 1:1000 |
| ErbB3 (WB); Rb | Cell Signaling (Beverly MA) | 1:500 |
| ErbB4 (WB); Rb | Cell Signaling (Beverly MA) | 1:1000 |
| ERK (WB); Rb | Cell Signaling (Beverly MA) | 1:1000 |
| FAK (WB); Rb | Cell Signaling (Beverly MA) | 1:1000 |
| GFAP (ICC); Rb | Sigma (St. Louis MO) | 1:500 |
| Merlin (WB); Rb | Cell Signaling (Beverly MA) | 1:1000 |
| mTOR (WB); Rb | Cell Signaling (Beverly MA) | 1:1000 |
| Non-phospho-β-catenin; Rb | Cell Signaling (Beverly MA) | 1:1000 |
| O4 (ICC); Ms IgM | Chemicon (Temecula CA) | 1:1000 |
| p-AKT (WB); Rb | Cell Signaling (Beverly MA) | 1:1000 |
| p-EGFR (WB); Rb | Cell Signaling (Beverly MA) | 1:1000 |
| p-ErbB2-Tyr1221/1222 (WB); Rb | Cell Signaling (Beverly MA) | 1:1000 |
| p-ErbB2-Y877 (IHC, WB); Rb | Abcam (Cambridge MA) | 1:50, 1:1000 |
| p-ErbB3 (WB), Rb | Cell Signaling (Beverly MA) | 1:500 |
| p-ErbB4 (WB); Rb | Cell Signaling (Beverly MA) | 1:1000 |
| p-FAK (WB); Rb | Cell Signaling (Beverly MA) | 1:1000 |
| p-ERK | Cell Signaling (Beverly MA) | 1:1000 |
| p-mTOR (WB); Rb | Cell Signaling (Beverly MA) | 1:1000 |
| p-SRC (WB); Rb | Cell Signaling (Beverly MA) | 1:1000 |
| p-YAP (WB); Rb | Cell Signaling (Beverly MA) | 1:1000 |
| Rac1 (WB); Ms | Millipore (Bedford MA) | 1:1000 |
| SRC (WB); Rb | Cell Signaling (Beverly MA) | 1:1000 |
| Tubulin (WB); Rb | Sigma (St. Louis MO) | 1:1000 |
| Tuj1 (ICC); Ms | Covance (Denver PA) | 1:1000 |
| YAP (WB); Rb | Cell Signaling (Beverly MA) | 1:1000 |

Immunocytochemistry (ICC), Immunohistochemistry (IHC) and Western Blot (WB), Ms: Mouse antibody; Rb: Rabbit antibody, Cl. = cleaved, p- = phospho-
